# Intraoperative mapping of executive function using electrocorticography for patients with low-grade gliomas

**DOI:** 10.1007/s00701-020-04646-6

**Published:** 2020-11-22

**Authors:** Yaara Erez, Moataz Assem, Pedro Coelho, Rafael Romero-Garcia, Mallory Owen, Alexa McDonald, Emma Woodberry, Robert C. Morris, Stephen J. Price, John Suckling, John Duncan, Michael G. Hart, Thomas Santarius

**Affiliations:** 1 Medical Research Council, Cognition and Brain Sciences Unit, University of Cambridge, 15 Chaucer Road, Cambridge, CB2 7EF UK; 2 Neurophys Limited, Cambridge, UK; 3 Department of Psychiatry, University of Cambridge, Cambridge, UK; 4 Department of Neurosurgery, Cambridge University Hospitals NHS Foundation Trust, Cambridge, UK; 5 Department of Neuropsychology, Cambridge University Hospitals NHS Foundation Trust, Cambridge, UK; 6 Division of Neurosurgery, Department of Clinical Neurosciences, University of Cambridge, Cambridge, UK; 7 Behavioural and Clinical Neuroscience Institute, University of Cambridge, Cambridge, UK; 8 Cambridge and Peterborough NHS Foundation Trust, Cambridge, UK; 9 Department of Experimental Psychology, University of Oxford, Oxford, UK; 10 Department of Physiology, Development and Neuroscience, University of Cambridge, Cambridge, UK

**Keywords:** Executive function, Low-grade glioma, Brain tumor, Functional mapping, Electrocorticography (ECOG), Patients, Awake neurosurgery, Intraoperative

## Abstract

**Background:**

Intraoperative functional mapping with direct electrical stimulation during awake surgery for patients with diffuse low-grade glioma has been used in recent years to optimize the balance between surgical resection and quality of life following surgery. Mapping of executive functions is particularly challenging because of their complex nature, with only a handful of reports published so far. Here, we propose the recording of neural activity directly from the surface of the brain using electrocorticography to map executive functions and demonstrate its feasibility and potential utility.

**Methods:**

To track a neural signature of executive function, we recorded neural activity using electrocorticography during awake surgery from the frontal cortex of three patients judged to have an appearance of diffuse low-grade glioma. Based on existing functional magnetic resonance imaging (fMRI) evidence from healthy participants for the recruitment of areas associated with executive function with increased task demands, we employed a task difficulty manipulation in two counting tasks performed intraoperatively. Following surgery, the data were extracted and analyzed offline to identify increases in broadband high-gamma power with increased task difficulty, equivalent to fMRI findings, as a signature of activity related to executive function.

**Results:**

All three patients performed the tasks well. Data were recorded from five electrode strips, resulting in data from 15 channels overall. Eleven out of the 15 channels (73.3%) showed significant increases in high-gamma power with increased task difficulty, 26.6% of the channels (4/15) showed no change in power, and none of the channels showed power decrease. High-gamma power increases with increased task difficulty were more likely in areas that are within the canonical frontoparietal network template.

**Conclusions:**

These results are the first step toward developing electrocorticography as a tool for mapping of executive function complementarily to direct electrical stimulation to guide resection. Further studies are required to establish this approach for clinical use.

## Introduction

Surgical resection for diffuse low-grade gliomas (dLGG) involves a fine balance between maximal extent of resection and preserving cognitive function [1]. A prominent effort in the field in recent years has been dedicated to extending the range of cognitive domains that are functionally mapped intraoperatively to optimize this onco-functional balance [2, 3]. In many centers, intraoperative functional mapping of some aspects of language is already done as part of standard care in an awake procedure using direct electrical stimulation (DES), in addition to mapping of motor and sensory pathways. Several other tasks have been proposed for the mapping of a range of cognitive domains, such as visuospatial functions, verbal memory, calculation, dual tasking, and executive function (EF) [4–10]. Among these, the mapping of EF is particularly challenging, and only a handful of studies are reported in the literature. These studies used DES with the Stroop task that tests for one aspect of EF, namely, conflict monitoring [8–10]. Wager et al. [10] reported effects on performance in the Stroop task when DES was applied on the anterior cingulate cortex. Puglisi et al. [8, 9] found that DES led to impairments in the Stroop task intraoperatively only when subcortical sites were stimulated, but not cortical ones. These DES-positive sites were confined to white matter tracts located under the inferior and middle frontal gyri. Furthermore, they reported that patients who underwent intraoperative mapping with the Stroop task had less EF deficits on average compared with a control group following surgery, most in verbal fluency. Nevertheless, beyond this sparse evidence, no established tests for mapping of EF have been proposed.

Several considerations may limit the ability to map EF intraoperatively. First, EFs are inherently complex, referring to a wide range of functions such as attention, reasoning, dual-tasking, conflict monitoring, hierarchical planning, and allocation of resources. Given the time constraints during surgical procedures and the complexity of intraoperative testing, it is impossible to test for all these aspects of EF. Second, it is widely accepted that EF is supported by a distributed network of regions across the frontal and parietal cortices. However, the detailed role of each of the regions or sub-networks within this network is not yet known. Third, unlike the primary motor cortex, for example, which is anatomically well-defined, the neuroanatomical markers for the frontoparietal network are only loosely defined and cover large cortical areas that may be hard to identify in full during surgery.

Robust evidence from studies that use functional magnetic resonance imaging (fMRI) data in healthy participants reveal consistent recruitment of a frontoparietal cortical network involved in control processes. This network is recruited when task difficulty level increases across a wide range of cognitive domains, such as spatial working memory, verbal working memory, maths, and multisource response interference, and has been termed the “multiple demand” (MD) network [11–14]. This network shows similar activation patterns across the two hemispheres and includes an anterior-posterior axis along the middle frontal gyrus (MFG), the posterior dorso-lateral frontal cortex (pdLFC), the anterior insula and adjacent frontal operculum (AI/FO), the pre-supplementary motor area (pre-SMA) and dorsal anterior cingulate cortex (preSMA/ACC), and the intraparietal sulcus (IPS) in the parietal lobe. Similar regions have been reported to be involved in multiple aspects of cognitive control across many other neuroimaging studies [15–21].

Task-based fMRI can be used to functionally identify the MD network in individual participants. In this procedure, neural activity evoked by a given task (e.g., spatial working memory) is contrasted with an easier version of the same task. Such contrast allows for manipulating task difficulty while controlling for other task factors such as stimuli, responses, and duration of trials. While the group-level activation map is highly consistent, substantial differences in activity patterns of individuals are observed [14, 22], demonstrating that neuroanatomical markers are insufficient to reliably identify the control network in individuals. Furthermore, at the individual participant level, MD regions in the frontal cortex can be dissociated from adjacent areas involved in language processing [23, 24].

Here, we propose a novel approach complementary to DES for intraoperative mapping EF using recordings of activity directly from the surface of the brain using electrocorticography (ECOG). DES and non-stimulatory ECOG data provide different types of information. To ascertain that a stimulated brain area/locus is subservient to EF is time consuming, which is a significant constraint in a time-limited surgery. Functional mapping using ECOG data, either from the surface of the brain or using depth electrodes, has the potential to narrow down the areas to subsequently focus on using DES. We build on neuroimaging evidence and use a similar task difficulty manipulation to identify brain areas that are associated with EF using ECOG. As demonstrated using fMRI, increased task difficulty leads to increased recruitment of attentional resources and therefore increased activity in the control network. By using such task difficulty manipulation, this approach probes the recruitment of networks involved in multiple core aspects of EF rather than tapping on a particular aspect of EF. Adapting tasks to the intraoperative setting, we used two counting tasks with varying difficulty levels: counting from 1 to 20 (simple counting, easy task) and counting while switching between letters and numbers 1-a-2-b-3-c… (switch counting, hard task) [25]. As a first step to investigating the utility and feasibility of this approach, functional measures of the recorded ECOG signals were analyzed offline and contrasted between the two tasks to identify a neural signature associated with increased task difficulty. Based on previously demonstrated links between the fMRI BOLD response and power in the broadband high-gamma frequency range (70–250 Hz) [26–28], we predicted that increased task difficulty will be associated with increases in high-gamma power in the recorded ECOG signals. In this paper, we describe this approach in detail and how it can be implemented in the operating theater. We then demonstrate the potential of its use in three patients. Finally, we discuss the steps required to further establish the validity of this approach and its real-time clinical use, intraoperatively.

## Methods and materials

### Patients

**Fig. 1:**
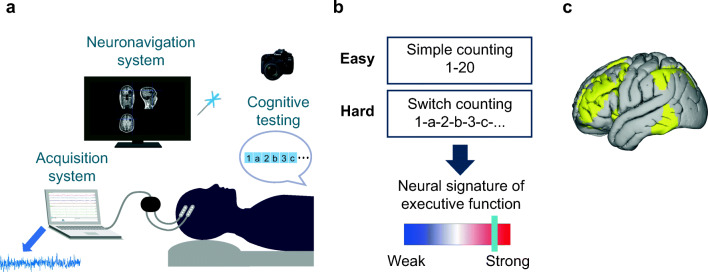
Intraoperative setup and cognitive tasks. **a** Intraoperative setup. Following the opening of the skull and dura, four-contact electrodes are placed on the surface of the brain. The electrodes are connected to an amplifier and the acquisition system, and local field potentials are recorded while the patient performs the counting tasks. The neuronavigation system and probe are used to register contact locations, and a picture of the craniotomy is taken to assist with the anatomical localization. The data was exported from the acquisition system following surgery and was analyzed offline. **b** Cognitive tasks. The patients performed 3–4 trials of each of the two counting tasks. The easy task was counting from 1 to 20 (simple counting). The harder task was counting while switching between letters and numbers (switch counting: 1-a-2-b-3-c…). Power in the high-gamma frequency range (70–250 Hz) was used to contrast activity between the hard and easy tasks, with increases in power with increased task difficulty attributed to the recruitment of areas associated with EF. **c** The canonical template of the fronto-temporo-parietal network (FTPN) as defined using resting-state fMRI in the study by Yeo et al. (2011)

The study was carried out at Addenbrooke’s Hospital, Cambridge, UK. Three patients with dLGG, in whom an awake craniotomy and some exposure of frontal lobes were anticipated, were included. The patients were evaluated by the neuro-oncology multidisciplinary team and were judged to have an appearance of a dLGG. Task-tailored awake craniotomy was used, as this is our method of choice for resection of dLGGs. The patients had no history of severe head injury or cranial radiotherapy. The patients gave written informed consent to participate in the study prior to surgery and were explicitly informed that the study will not have any implications for their clinical care and treatment. The study was approved by the East of England—Cambridge Central Research Ethics Committee (REC reference 16/EE/0151). The extent of craniotomy for all patients was determined by clinical considerations to allow optimum resection of the tumor. For all patients, the craniotomy extended to the frontal lobe, allowing the placement of electrodes for ECOG recordings (Fig. 1a).

### Neuroimaging

Magnetic resonance imaging (MRI) data were acquired pre-operatively using a Siemens Magnetom Prisma-fit 3 Tesla MRI scanner and a 16-channel receive-only head coil (Siemens AG, Erlangen, Germany). Structural anatomic images were acquired using a T1-weighted magnetization prepared rapid gradient echo (MPRAGE) sequence (field of view 256 mm × 240 mm × 176 mm; voxel size 1 mm isotropic; time to repetition (TR) 2300 ms; time to echo (TE) 2.98 ms; flip angle 9°). The acquisition time was 9 min and 14 s.

### Intraoperative cognitive tasks

The choice of tasks was based on robust and consistent evidence from fMRI studies with healthy participants showing recruitment of EF-related regions with increased task difficulty across a range of cognitive domains [11–14]. We adopted tasks used in fMRI experiments to recruit the control frontoparietal network to testing in the operating room and used two tasks with a manipulation of difficulty level, with one task being easy and the other being harder (Fig. 1b). Each patient performed two short tasks with 4–6 repetitions of each while alternating between them. The tasks were designed to be appropriate for the patients to perform during surgery and short so they can be repeated several times. In the easy task (simple counting), the patients counted from 1 to 20. In the hard task (switch counting), the patients counted while switching between numbers and letters (1-a-2-b-3-c and so on). This task also included a task-switching aspect and shares some features with the trail task [29] that is commonly used to assess EF as part of standard neuropsychological assessments. To allow for experimental control beyond the difficulty level manipulation, the tasks were designed to be as matched as possible across the following factors: have similar cognitive components (i.e., counting), have similar presentation form (verbal instructions, no fast-moving visual input as usually used in equivalent fMRI experiments), and require the same response modality (i.e., verbal). Therefore, changes in activity evoked by the harder task compared with the easier one reflect the recruitment of areas/loci associated with EF with increased difficulty level and serve as a neural signature of EF-related activity while dissociating it from other factors that are controlled across the two tasks, such as speech production. Overall, the recording time was 7–9 min, including brief intervals between the tasks to inform the patient about the next task, with an additional 5–10 min required for setup and electrode registration (see below).

### Data acquisition

Following opening of the skull and dura mater, patients were awakened, and cognitive testing was performed under fully awake conditions prior to any resection. For each patient, 1–2 strip electrodes with 4 contacts each were temporarily placed on the cortical surface on a tissue that was judged by the neurosurgeon to be healthy, based on imaging and intraoperative findings. Electrodes were either straight (MS04R-IP10X-0JH, Ad-Tech, Medical Instruments corporation, WI, USA) or T-shaped (CORTAC 2111-04-081, PMT Corporation, MN, USA). For both electrode types, contact-to-contact spacing was 10 mm from the center of contact. Contact disks were 5 mm and 3 mm in diameter for the straight and T-shaped electrodes, respectively. Electrodes were placed further away from the tumor such that the recorded data could be attributed to normal neural activity rather than the abnormal one as a result of close proximity to a diffuse tumor. Electrode impedance was 1.5–4.5 KΩ. Data were recorded using a 32-channel amplifier (Medtronic Xomed, Jacksonville, FS, USA) sampled at 10 KHz while the patients performed the tasks. Markers for the start and end of performance of each task were entered manually into the acquisition system. Potential sources of electrical noise such as microscope, patient warming blanket, and IV pumps were identified and repositioned in order to avoid signal contamination. The data were recorded via dedicated channels on the acquisition system and was band-pass filtered at 1 Hz and low-pass filtered at 1500 Hz using a Butterworth filter. A ground needle electrode was connected to the deltoid muscle, and the electrodes were referenced to a mid-frontal (Fz) spiral scalp EEG electrode. The electrodes were removed after the recording has been completed. Following surgery, data were extracted from the acquisition system for offline analysis.

### Electrode localization

For patient 2, contact coordinate data were registered intraoperatively using a neuronavigation system (StealthStation® S7® System, Medtronic, Inc., 24 Louisville, CO, USA). The stereotactic probe was placed on the center of each contact, and coordinate data were registered and saved in the neuronavigation system. Stereotactic snapshots of contact positions were taken using the neuronavigation system to further assist with the spatial localization of the electrodes. After surgery, coordinate data as registered intraoperatively were further verified by visualizing them on each patient’s preoperative structural MRI scan using the Oxford Centre for Functional MRI of the Brain (FMRIB) FSLEyes software (https://fsl.fmrib.ox.ac.uk/fsl/fslwiki/FSLeyes) and then matching them with the stereotactic snapshots taken intraoperatively. To allow the rendering of contact locations on a common brain template, the pre-operative T1w image of the patient was co-registered with the standard MNI brain template (MNI152) at 2 mm resolution. The Oxford Centre for Functional MRI of the Brain (FMRIB) Linear Image Registration Tool (FLIRT) was used for co-registration with 12° of freedom (full set of affine transformations) and correlation ratio cost function. The coordinate data of each contact in native space were then multiplied by the resulting subject-to-MNI transformation matrix to determine the location of the contacts in MNI152 space.

For patients 1 and 3, a grid approach was used for electrode localization [30, 31]. Grids were drawn on a photograph of the brain taken during surgery and on the extracted template surface, and the contacts as they appeared on the brain photograph were localized on the template relative to the grid. First, visible major sulci were delineated on the intraoperative craniotomy photographs: precentral sulcus, Sylvian fissure, and inferior and superior frontal sulci. Spaces between these sulci were populated by lines (1.5 cm apart) from the precentral sulcus toward the prefrontal pole to create a grid-like structure. Next, a grid was created in the same way for a template cortical surface. The template cortical surface was extracted by using FreeSurfer (https://surfer.nmr.mgh.harvard.edu) to reconstruct the surface of the MNI space 0.8-mm isotropic volumetric map. The location of each contact was then extracted by manually marking its approximate location on the MNI cortical grid while it was visualized using Connectome Workbench (version 1.4.2, https://www.humanconnectome.org/software/get-connectome-workbench). To transform contact locations from the surface to a 3D volumetric space, the MNI volumetric map was co-registered with its surface reconstruction in Connectome Workbench. This allowed for the automatic transformation of any point marked on the surface back to its 3D volumetric MNI coordinates.

To assist with the anatomical localization, photographs of the craniotomy with the placed electrodes were taken during surgery using either a dedicated camera (Sony DSLR-A200K, Sony) (patients 1 and 2) or a microscope (ZEISS OPMI PENTERO 800, Carl Zeiss AG) (patient 3). Electrode locations were verified using the photographs based on anatomical features (gyri, sulci, and vascular anatomy) and the pre-operative MRI scan. Electrode displacements due to brain shifts (caused by pressure changes related to craniotomy) were compensated by back-projecting the coordinates onto the cortical surface along the local norm vector [32] as implemented in the FieldTrip protocol for human intracranial data [33].

### Data analysis

The continuous time-series recorded intraoperatively was imported to Matlab (v2018a) and analyzed using EEGLAB (v13.6.5b) and custom Matlab code. Preprocessing included:

1. Down-sampling to 2 kHz.
2. Re-referencing using a bipolar scheme: each channel was re-referenced to an adjacent channel from the same electrode (1 minus 2, 2 minus 3, 3 minus 4). This resulted in data from three channels for each electrode that was further analyzed.
3. Noise filtering was applied using a notch filter at 50 Hz and its harmonics. The notch filter was also applied at 79 Hz and its harmonics to remove additional noise that was observed in the data due to equipment in the operating theater.
4. The data were band-pass filtered to include the broadband high-gamma frequency band (70–250 Hz).
5. The instantaneous power of the time-series was computed using the square of the absolute amplitude envelope of the Hilbert transformed data.

Quality checks for all channels were conducted during preprocessing and prior to any further analysis to ensure that the signals are suitable for subsequent analysis, including inspection of the amplitude and variance distribution as well as the power spectral density function. Only patients with good quality of the signals were included in this study. Objective quality assurance methods will have to be developed in future studies for this method to be informative to the surgeon during an operation.

Following preprocessing, data were segmented into separate conditions and trials. To account for potential temporal inaccuracies of onset and offset markers (e.g., when the patient started counting slightly after the “go” signal or due to the manual insertion of markers into the acquisition system), 1 s from the beginning and end of each trial was excluded. For the switch counting trials (hard condition), an additional 3 s were excluded from the beginning of each trial to account for the part of the trial in which counting was easier (1-a-2-b-3-c…) and ensure that the more demanding part of the trial was used for analysis. The mean high-gamma power for each condition (simple counting, switch counting) was then computed by concatenating the data of all time points across trials and averaging the instantaneous power across all time points. The proportion of change in power between the hard (switch counting) and easy (simple counting) conditions was computed by dividing the mean power of the former by the mean power of the latter. The percentage of signal change (PSC) was then computed by subtracting 1 (equal power for both conditions) and multiplying by 100 (converting proportion to percentages). Positive PSC indicates an increase in power for the hard vs. easy condition, and a negative PSC indicates a decrease in power.

To provide per-channel statistical inference for power modulations between the two conditions, we used a permutation approach. For each channel, instantaneous power from all trials of the two conditions was concatenated serially. The end of the last trial was circularly concatenated to the start of the first trial to form a loop of instantaneous power data across all trials. A random jitter was then used to shift all trial onset and offset markers and “rotate” them along the trial data loop. Using this “rotation” method enabled the creation of surrogate trials for the two conditions while maintaining all block lengths and preserving the correlation structure of the data. The mean power for each condition based on the surrogate trials was computed, followed by computing new PSC values. This was repeated 100,000 times to create a null distribution of power modulations. The PSC value was then compared against this distribution using a two-tailed statistical threshold with a type I error rate, alpha = 0.05. A PSC value was considered as statistically significant if it was larger than a percentile rank of 97.5% (increase in power for hard vs. easy) or smaller than a percentile rank of 2.5% (decrease in power for hard vs. easy).

To relate channel locations to areas that may be more likely to be associated with EF, we used the canonical fronto-temporo-parietal network (FTPN) as defined by prior resting-state fMRI data (Yeo et al., 2011) (Fig. 1c). The volumetric mask of the FTPN was resampled to 2-mm MNI space to match the resolution of electrode locations in the same space. To ensure that the FTPN mask covered the surface of the brain where the channels were located, all voxels with probability greater than 0 following the resampling process were included in the mask. We then determined for each channel whether it resided within or outside this mask based on the channel coordinate data in MNI space.

## Results

We demonstrate the feasibility of using ECOG for the intraoperative mapping of EF in three patients with diverse clinical profiles. The clinical care and surgery of the patients was not affected by the electrophysiological data and EF mapping presented here. Data were recorded from five electrode strips with four contacts each placed on the frontal cortex, resulting in data from 15 channels overall following bipolar re-referencing. Eleven out of the 15 channels (73.3%) showed significant increases in high-gamma power with increased task difficulty, 4 channels (26.6%) showed no change in power, and none of the channels showed a power decrease. The increases in power were consistent with our prediction and suggest that the recorded areas are associated with EF more generally. Overall, changes in high-gamma power with increased task difficulty were in partial overlap with the FTPN template, with increases being more likely in areas that were within the FTPN template and less likely outside it. Out of the 11 channels that showed power increases, 9 (81.8%) were located within the FTPN template, and only 2 (18.2%) were located outside the template. Of the 4 channels that showed no change in power, 3 (75%) were located outside the FTPN template and only 1 (25%) within the FTPN. These results provide support for the link between the observed changes in high-gamma power and EF-related regions. Below, we describe in detail the clinical profile and electrophysiology results for each of the three patients.

### Patient 1

**Fig. 2:**
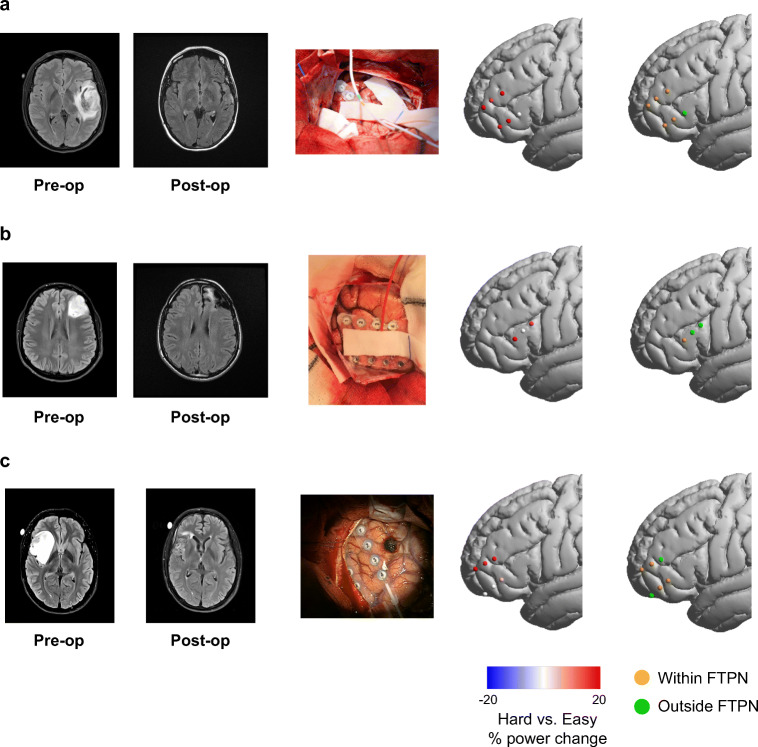
Increase in high-gamma power with increased task difficulty in the frontal cortex. **a** Data for patient 1. From left to right: pre- and post-operative MRI scans; craniotomy showing the electrodes placed on the surface of the brain; channels rendered on a template brain showing the percentage of high-gamma power change with increased task difficulty (switch counting vs. simple counting). Each dot is a channel, with red and blue showing significant power increase and decrease, respectively. Percentage of power change beyond ± 20% is set to ± 20%. Channels that did not show significant power changes are shown in white. The rightmost column shows channels rendered on a template brain with colors indicating channels within (orange) and outside (green) the FTPN template. For visualization, all channels are shown on the left hemisphere. **b** Results for patient 2. Another electrode was placed on the tumor. We focus here on the activity in intact areas that can be appropriately interpreted with respect to the cognitive tasks; therefore, data from this electrode were not analyzed. **c** Results for patient 3. Details for patients 2 and 3 are the same as in **a**

The patient was a 33-year-old right-handed female who had an MRI scan as a part of an investigation for headaches. The MRI revealed a non-enhancing left temporal tumor consistent with a dLGG (Fig. 2a, left). Following surgery, the tumor was diagnosed as an anaplastic astrocytoma, IDH-mutant (WHO grade III).

Pre-operatively, the patient scored with the expected range or higher on all neuropsychological tests including attention and EF. At 2 weeks and 6 months post-surgery, the patient scored comparably with baseline assessments on EF tasks and showed mild improvement in attention tests.

The craniotomy partially exposed the frontal cortex (Fig. 2a, middle), and two straight electrodes were placed on the middle and inferior frontal gyrus. The patient performed very well in the counting tasks and conducted 4 interleaved trials of each task (mean ± SD 17.8 ± 6.1 s and 27.3 ± 4.8 s for the simple and switch counting, respectively), with a total testing time of 7 min.

Broadband high-gamma activity during task performance showed significant increases in power with increased task difficulty (switch vs. simple counting) in all three channels on the middle frontal gyrus, and two of the three channels on the inferior frontal gyrus, with PSC increases ranging between 14.5 and 27.1% (mean ± SD 21.7% ± 4.9%, *p* < 0.02 for all channels) (Fig. 2a, right). The most posterior channel on the inferior frontal gyrus did not show a significant PSC (*p* = 0.6), and negative PSCs were not observed in any of the channels. All five channels that showed increases in high-gamma power with increased task difficulty were located within the canonical FTPN template, and the single channel that showed no changes in power was located outside this template.

### Patient 2

The patient was a 29-year-old right-handed male that presented with a seizure, and an MRI scan revealed a non-enhancing tumor in the left middle frontal gyrus consistent with a dLGG (Fig. 2b, left). The tumor was later diagnosed as a diffuse astrocytoma WHO grade II (ADH1-mutant, ATXR mutated, 1p-19q non-codeleted).

Pre-operative neuropsychological assessment showed no impairment of EF or attention. Performance at a 2-week post-surgery assessment showed mild deterioration of EF related to an inhibition task and a mild increase in performance on an initiation task when this was not timed. Attention scores were comparable with pre-operative baseline. On a 6-month follow-up assessment, the patient performed well on tests of attention and EF; however, he reported some reduction in EF on tasks when quick brain processing was required.

During surgery, following craniotomy and awakening of the patient from anesthesia, and prior to any resection, a T-shaped electrode was placed on the inferior frontal gyrus (Fig. 2b, middle). ECOG data were recorded during 6 simple (mean ± SD 17 ± 3.4 s) and 4 switch counting (mean ± SD 23.8 ± 5 s) interleaved trials, with a total testing time of 7 min.

Electrophysiology data showed a significant increase in high-gamma activity with increased task difficulty in two out of the three channels (Fig. 2b, right). High-gamma activity in the most anterior channel had a PSC of 20% (*p* = 0.0009), and this channel was located within the FTPN template. Increase in high-gamma activity was also observed in the most posterior channel (PSC = 14%, *p* = 0.047), although it was located outside the FTPN template. The middle channel was also located outside the FTPN template and showed no significant change in high-gamma PSC (*p* = 0.12).

### Patient 3

The patient was a 31-year-old right-handed male presented with nocturnal seizures, and an MRI scan demonstrated a right, predominantly insular tumor (Fig. 2c, left). The tumor was diagnosed post-surgery as a grade II diffuse astrocytoma, IDH1-wild type, ATRX-deleted.

Pre-operative neuropsychological assessment showed performance in attention and EF tests mostly within the expected range, with only mild impairments in a two-digit span verbal attention test, which may have been related to the patient’s difficulty with numbers because of dyslexia. In a 2-week post-surgery neuropsychological assessment, the patient performed at a level similar or better than the pre-operative assessment on attention and EF tests. Although the patient did not notice any cognitive changes or difficulties following surgery, his partner noticed that his processing speed was slightly slower in more distracting environments. In a 6-month follow-up assessment, the patient performed the same or better compared with pre-operative baseline on all attention and EF tests.

During surgery, the patient was awakened, and two straight electrodes were placed on the middle and inferior frontal gyrus (Fig. 2c, middle). ECOG data was recorded prior to any resection during, 5 trials of the simple counting task and 4 trials of the switch counting task (mean ± SD 21.2 ± 7.8 s and 35.8 ± 7 s, respectively), with a total recording time of 9 min.

Four of the six channels showed significant increases in high-gamma power with increased task difficulty (Fig. 2c, right). All three channels located on the middle frontal gyrus showed significant increases of high-gamma power with PSC ranging between 16.8 and 19.8% (*p* < 0.008, for all three channels). Out of these, the two anterior contacts were located within the FTPN template, and the most posterior one was located outside. Only the most posterior contact on the inferior frontal gyrus had a significant increase in high-gamma power (PSC = 4.7%, *p* = 0.033), although moderate, and was located within the FTPN template. The two other contacts on the inferior frontal gyrus did not show significant changes in high-gamma power (*p* > 0.28).

## Discussion

We developed a novel approach for intraoperative functional mapping of EF using ECOG and demonstrated its feasibility and applicability in three patients undergoing awake surgery for the resection of low-grade glioma. This approach is based on the rationale of recruiting areas that are associated with EF by manipulating task demands. We described in detail the procedure and implementation of this approach and showed that increases in broadband high-gamma power with increased task difficulty in two counting tasks may be related to areas that are associated with EF. We emphasize the importance of intraoperative testing for EF and propose that electrophysiological signals may be used intraoperatively complementarily to DES to guide and make the functional procedure more effective, efficient, and fast. Our results serve as “proof of concept,” and future research will be required to establish its validity and benefit in clinical practice.

We demonstrated the use of this approach in three patients with diverse clinical and cognitive characteristics. Patient 1 had a temporal tumor, away from the recording locations in the frontal lobe; therefore, electrophysiology activity likely reflects normal responses to the task difficulty manipulation, within surgical limitations and possible anesthetic effects. Indeed, five out of the six channels showed the expected electrophysiological signature of EF in the form of increases in high-gamma power with increased task difficulty, and these channels were located within the FTPN template. The only channel that did not show such an increase was located outside the FTPN template. None of the recording areas was resected or in proximity to the resected tissue in the temporal lobe, and EF and attention performance following surgery were preserved, or even mildly improved. While this is in line with evidence for the role of frontal and parietal areas in EF, it has been recently shown that the temporal lobe is implicated specifically in set-shifting during a trail making test [34, 35], and further studies will be required to clarify the involvement of the temporal lobe in EF. The case of patient 2 demonstrates the potential benefit of intraoperative EF monitoring using ECOG in areas around the tumor for preventing post-surgery EF deficits while maximizing the extent of resection. The tumor was located in the middle frontal gyrus, an area that is associated with EF and attentional processes. The tissue at the vicinity of the tumor, where resection could extend to, may also be involved in such cognitive functions, either because it was involved in EF before the tumor developed and/or because of plasticity processes and potential migration of functionality due to tumor growth. Therefore, mapping EF in areas adjacent to the tumor could contribute to refining the resected areas to preserve EF following surgery. Indeed, although the patient did not have EF or attention impairments prior to surgery, such deficits were identified in the post-surgery assessment and were self-reported in the 6-month follow-up appointment. Electrophysiology data were recorded from the inferior frontal gyrus in areas largely considered as part of the EF network and in partial overlap with the resting-state FTPN and showed increases in high-gamma power with increased task difficulty in some of the channels. Possibly, areas in close proximity to the tumor and within the EF-related network exhibit similar response profiles that could be identified using ECOG. In patient 3, the recording locations were mostly areas that are classically associated with EF. The insular tumor was further away from the areas where electrophysiology data was recorded, suggesting that anatomo-functional relations in the frontal cortex are unlikely to change because of tumor growth and that the neural activity recorded from the frontal cortex likely reflects activity in normal tissue, in line with the results. However, the insula is considered as part of the EF/control network, and possible impairment of the FTPN during the resection of the insula may have contributed to the mild deficits in processing speed that were self-reported by the patient’s partner following surgery.

The intraoperative mapping of EF is considered to be challenging and difficult and has been so far addressed in only a handful of studies using DES with Stroop or trail making tasks as single aspects of EF [8–10, 35]. We showed that using counting tasks with increased difficulty while recording activity directly from the surface of the brain using ECOG is a feasible approach that can be conducted intraoperatively. At the behavioral level, patients were able to perform the tasks well. For the functional measures of the recorded brain signals, we build on previous evidence from fMRI in healthy participants that show consistent and robust recruitment of the frontoparietal control network when task demands increase [11, 14, 20, 22]. Rather than a specific aspect of EF, our approach focuses on the recruitment of the control network as has been observed in neuroimaging studies. Supported by ample evidence, this network has been associated with a variety of cognitive functions associated with EF and across multiple cognitive domains. These include working memory, maths, conflict monitoring, complexity, and representation of behaviorally relevant information such as context, stimulus information, and decisions [14, 18–21, 36, 37]. In accordance with these neuroimaging findings, we used a similar task difficulty manipulation and measured changes in intracranial EEG signals. We focused on power in the broadband high-gamma frequency range (70–250 Hz), which has been previously linked to the fMRI BOLD response [26–28]. Power increases in the gamma band have been associated with a range of cognitive functions including attention, working memory load, rule abstraction, and stimulus processing [38–43]. Measures of power in the gamma range have been used to control movement in brain-computer interfaces [44, 45] as well as for mapping in the motor and language domains [46–49]. Here, we demonstrated that power increases in the gamma range are also observed for increased task difficulty in the frontal cortex, and these can be measured using ECOG during an awake surgery procedure, serving as an approximate measure for the recruitment of the control network.

In this study, we recorded the data intraoperatively and analyzed it offline in order to establish the feasibility and utility of this approach. Ultimately, the aim is to provide neurosurgeons with feedback in real time during surgery to identify areas that are most likely to be associated with EF and guide the use of DES toward these areas. Moreover, DES and ECOG data measure different neural responses. DES provides information about the causal role of the stimulated area in the tested function and the potential outcome of resection. In contrast, the electrophysiological signal as measured with ECOG provides evidence for the involvement of the recorded area in a certain function but not if it is essential for this function. While DES serves as the gold standard in awake function mapping, the ability to detect effects of stimulation depends on multiple factors, which may make it challenging for EF in particular. The applied current intensity is usually determined based on motor threshold and may vary between brain regions which may require a higher intensity (and potentially unsafe) to interrupt behavioral responses. For example, effects of DES on performance in the Stroop task have been demonstrated when white matter tracts were stimulated, but not gray matter tissue [8, 9], in line with the higher excitability of white matter [50]. Conversely, a larger area of the brain may be stimulated with higher current settings. Additionally, behavioral performance in some cognitive functions such as EF may depend on a distributed network of regions rather than a focal area that can be stimulated. Therefore, DES of a single area might not result in detectable behavioral impairments intraoperatively, yet post-surgery outcomes may show cognitive deficits with potentially impactful effects on quality of life of patients. Indeed, it has been demonstrated that partial damage to the frontoparietal control network in stroke patients is associated with reduced cognitive ability as measured with IQ [51]. Finally, because of the complex nature of EF, DES that is used in conjunction with a specific task, such as the Stroop that reflects one aspect of EF, may not generalize to other aspects of EF. Altogether, despite the clear benefits of DES in clinical practice for the standard mapping of motor and language-related functions, its use for mapping of EF poses substantial difficulties that may result in some EF-related areas that are missed out. Using ECOG to guide and complement DES may provide useful information to further improve and optimize the functional mapping procedure, and this was the primary clinical motivation for this study. We note, however, that despite the additional information that ECOG may offer, this technique is not suitable for monitoring EF in white matter, in contrast to DES [8, 9, 35]. We emphasize that using ECOG for mapping EF during awake surgery, as we demonstrate here, is not intended to be a substitute for DES mapping. DES remains the gold standard for deciding whether any brain tissue should or should not be removed. ECOG data acquired while patients perform a task may provide important information that is needed to preserve certain functions, such as EF, that cannot be readily assessed in a few seconds following DES. Here, we identified an ECOG signal associated with EF, providing initial evidence that can be further pursued in the field.

The data reported here serve as the first step toward developing ECOG as a tool for intraoperative mapping of EF. We demonstrated the feasibility of this approach, but a careful and rigorous evidence-based investigation at multiple levels is required to further validate and establish this approach and its clinical utility before it can be used routinely. First, a larger sample size and better coverage of the frontal cortex is necessary to provide solid evidence for increases in high-gamma activity with increased demand in the frontal cortex. Linking such increases as measured with ECOG to evidence of frontal activity from other modalities such as neuroimaging will further strengthen such evidence. Specifically, investigating the relationship between ECOG measures and task-based fMRI BOLD response associated with EF may contribute to better understanding of the potential and limitations of using fMRI for pre-operative planning and more generally inform about how the two signals relate to one another. Importantly, conjunctive evidence from DES and ECOG will shed light on the relationship between the two and, in particular, whether areas with increased high-gamma activity when task demands increase as measured by ECOG indeed show higher probability of exerting DES effects. Increases in high-gamma activity with increased task demands may also be related to post-surgery EF deficits, for example, in resected areas adjacent to the tumor. An essential step in bringing our proposed technique into use in clinical practice would involve establishing the importance of preserving areas associated with EF to long-term clinical outcome [52]. While deficits associated with EF following lesions in fronto-parietal areas have been demonstrated in patients with stroke [51], in patients with dLGG, there is remarkable long-term recovery through network reorganization and plasticity following surgery, which is not yet well understood. Ultimately, a controlled trial will be required to demonstrate the potential benefit of using ECOG for intraoperative mapping of EF, for example, in the form of a larger extent of resection and improved cognitive outcome post-surgery and as a result overall better quality of life of patients.

## Conclusion

We have shown the feasibility of intraoperative ECOG as a tool for mapping EF, and we believe that it has a potential to augment intraoperative functional brain mapping by guiding and supporting DES. While we used electrodes that were placed on the surface of the brain, similar data can be obtained using depth electrodes, offering access to deeper layers and structures before resection and the possibility of identifying their involvement in the tested cognitive functions. Beyond EF, intraoperative mapping using ECOG and in combination with DES offers opportunities for expansion to other cognitive domains, as part of the growing interest in individually tailored treatments that can be offered to patients.
